# Reduced Kidney Function Is Associated with Increasing Red Blood Cell Folate Concentration and Changes in Folate Form Distributions (NHANES 2011–2018)

**DOI:** 10.3390/nu14051054

**Published:** 2022-03-02

**Authors:** Arick Wang, Lorraine F. Yeung, Nilka Ríos Burrows, Charles E. Rose, Zia Fazili, Christine M. Pfeiffer, Krista S. Crider

**Affiliations:** 1 National Center on Birth Defects and Developmental Disabilities, Centers for Disease Control and Prevention, Atlanta, GA 30341, USA; lcy5@cdc.gov (L.F.Y.); cvr7@cdc.gov (C.E.R.); kvc3@cdc.gov (K.S.C.); 2 Division of Diabetes Translation, National Center for Chronic Disease Prevention and Health Promotion, Centers for Disease Control and Prevention, Atlanta, GA 30341, USA; nmr0@cdc.gov; 3 National Center for Environmental Health, Centers for Disease Control and Prevention, Atlanta, GA 30341, USA; zxq0@cdc.gov (Z.F.); cfp8@cdc.gov (C.M.P.)

**Keywords:** folate concentration, chronic kidney disease, folic acid, folate metabolism

## Abstract

Background: Current studies examining the effects of high concentrations of red blood cell (RBC) or serum folates assume that high folate concentrations are an indicator of high folic acid intakes, often ignoring the contributions of other homeostatic and biological processes, such as kidney function. Objective: The current study examined the relative contributions of declining kidney function, as measured by the risk of chronic kidney disease (CKD), and usual total folic acid intake on the concentrations of RBC folate and serum folate (total as well as individual folate forms). Design: Cross-sectional data from the National Health and Nutrition Examination Survey (NHANES) collected in 2-year cycles were combined from 2011 to 2018. A total of 18,127 participants aged ≥16 years with available folate measures, kidney biomarker data (operationalized as a categorical CKD risk variable describing the risk of progression), and reliable dietary recall data were analyzed. Results: RBC folate concentrations increased as CKD risk increased: low risk, 1089 (95% CI: 1069, 1110) nmol/L; moderate risk, 1189 (95% CI: 1158, 1220) nmol/L; high risk, 1488 (95% CI: 1419, 1561) nmol/L; and highest risk, 1443 (95% CI: 1302, 1598) nmol/L (*p* < 0.0001). Similarly, serum total folate concentrations increased as CKD risk increased: low risk: 37.1 (95% CI: 26.3, 38.0) nmol/L; moderate risk: 40.2 (95% CI: 38.8, 41.7) nmol/L; high risk: 48.0 (95% CI: 44.3, 52.1) nmol/L; the highest Risk: 42.8 (95% CI: 37.8, 48.4) nmol/L (*p* < 0.0001). The modeled usual intake of folic acid showed no difference among CKD risk groups, with a population median of 225 (interquartile range: 108–390) µg/day. Conclusion: Both RBC and serum folate concentrations increased with declining kidney function without increased folic acid intake. When analyzing associations between folate concentrations and disease outcomes, researchers may want to consider the confounding role of kidney function.

## 1. Introduction

Many scientific studies have been conducted on folate and its synthetic isoform—folic acid. Due to the central role of folates in the one-carbon metabolism pathway, folate deficiencies and insufficiencies are linked with a myriad of adverse health issues from birth defects to cancer; however, evidence of adverse effects of high folate intake or blood concentration is inconsistent [1]. Studies widely assume that high folate concentrations in the blood are a direct result of high folate intake without considering the complex interactions of homeostasis, specifically mediated by body use and excretion. The kidneys play two essential roles in folate metabolism: first, the secretion of metabolites, including folates used as reducing agents and folic acid, and waste from the blood into the urine; and second, renal reabsorption of folates to conserve and recycle them back into the bloodstream [2]. Changes in kidney function might lead to homeostatic changes in circulating folate concentrations independent of intake. Severe kidney dysfunction itself can create an inflammatory microenvironment that can be conducive to increased risk of several adverse outcomes such as cancer [3] or increased toxicity from the accumulation of waste and toxicants such as arsenic [4,5,6]. Thus, changes in circulating folate concentrations might be modified by homeostatic changes due to declining kidney function, and studies of high folate concentrations might have etiologies more closely linked to kidney disease. To better delineate the role the kidneys might play in folic acid metabolism and folate concentrations, we examined the relationship between available biomarkers of kidney function (urine and serum) and RBC and serum folate concentrations among adults aged ≥16 years using the National Health and Nutrition Examination Survey (NHANES) from 2011 to 2018.

## 2. Subjects and Methods

### 2.1. Demographic Characteristics

We used cross-sectional NHANES data from 2011 to 2018 using a stratified multistage probabilistic design that collected data in 2-years cycles: 2011–2012, 2013–2014, 2015–2016, and 2017–2018. Each 2-years NHANES cycle captured a nationally representative sample of the U.S. non-institutionalized civilian population. Respondents participated in household interviews and in-person physical examinations that included a blood draw and urine sample taken at a Mobile Examination Center (MEC). A 24 h in-person recall dietary interview was conducted at the MEC with a phone interview follow-up. Detailed descriptions of the survey have been published elsewhere [7,8,9,10]. The subpopulation for this analysis consisted of all non-pregnant people aged ≥16 who had available RBC and serum folate measures, available kidney biomarker data, and with dietary recall data labeled as “reliable and met minimum requirements” for at least the first of 2 dietary recalls. All adult participants in NHANES provided written informed consent and documented assent was obtained for children aged 16–17. Participant flowchart provided in Figure S1.

Information on age, sex, race/Hispanic origin, education, and poverty–income ratio (PIR) were obtained through a household questionnaire, and the body mass index (BMI) was obtained from the MEC. Age was stratified into 3 categories that allowed for adequate sample sizes across chronic kidney disease (CKD) risk categories: 16–59, 60–74, and ≥75. Education was collapsed into 3 categories: less than high school education, high school diploma or General Education Development (GED) diploma with some or no college, and at least a college degree. PIR was based on family reported household income and household size; PIR values of <1.0 are considered below the poverty threshold. Cells that contained fewer than 30 individuals were excluded from the analysis.

### 2.2. Kidney Biomarkers, Liver Biomarkers, and Blood Folate Concentrations

Urinary albumin concentrations were measured using a fluorescent immunoassay, and urine creatinine concentrations were assayed using an enzymatic method [11]. Urinary albumin-to-creatinine ratio (ACR, mg/g) was computed to determine albuminuria stages. Serum creatinine concentrations were assayed using the Jaffe rate method from 2011 to 2016 and using the enzymatic method from 2017 to 2018, which were adjusted to match prior cycles using regression equations [12]. Serum creatinine, measured using an enzymatic method, was used to calculate an estimated glomerular filtration rate (eGFR) using the CKD–EPI creatinine equation [13]. Calculated albuminuria and eGFR stages were used to form a composite chronic kidney disease (CKD) risk category, defined by the relative risk of CKD progression (an average annual decrease in eGFR of ≥2.5 mL/min per 1.73 m^2^ per year) by a composite score from a meta-analysis described by the Kidney Disease Improving Global Outcomes (KDIGO) Workgroup [14]. Alanine aminotransferase (ALT) and aspartate aminotransferase (AST) were both measured from serum using Roche Cobas 6000 model and Roche reagent kits (Roche Diagnostics, Mannheim, Germany) for ALT and AST, respectively.

RBC folates were measured using a microbiologic assay [11]. Adjusted geometric means (aGM) of RBC folates were calculated for each CKD risk group, taking BMI, PIR, and education into consideration. Five folate forms, 5-methyltetrahydrofolate (5-methylTHF), unmetabolized folic acid (UMFA), 5-formyltetrahydrofolate (5-formylTHF), tetrahydrofolate (THF), 5,10-methenyletrahydrofolate (5,10-methenylTHF), and pyrazino-s-triazine derivative of 4-α-hydroxy-5-methyltetrahydrofolate (MeFox), were measured in serum via liquid chromatography-tandem mass-spectrometry [11]; aGM for serum folate forms were calculated for each CKD risk group, adjusted for BMI, PIR, and education; three nonmethyl folate forms (5-formylTHF, THF, and 5,10-methenylTHF) were combined for analysis. Serum total folate concentrations consisted of all forms except MeFox. A ratio of RBC total folate concentration to serum folate concentration was calculated. Because age, sex, and race were used in the CKD-EPI creatinine equation for eGFR, no adjustments for these variables were made to the geometric mean.

### 2.3. Usual Intake

The usual intake of total folic acid was estimated using the National Cancer Institute (NCI) method comprising two 24 h dietary recalls and an average daily supplement for 30 days prior to dietary recalls [15]. A one-part model was used for the median and interquartile range (IQR) of folic acid consumed. Age, PIR, BMI, education, and race/Hispanic origin were used as covariates for the NCI model, accounting for weekend and weekday consumption. Additionally, food codes from the US Department of Agriculture were used to categorize subjects by folic acid intake source into four levels used in previous studies [16]: (1) those whose sole dietary folic acid was from background fortification of enriched cereal grain product (ECGP only), (2) those who ate ready-to-eat (RTE) cereals in addition to ECGP (ECGP + RTE), (3) those who took folic acid supplements (SUP) in addition to ECGP (ECGP + SUP), and (4) those who consumed folic acid from RTE cereals and supplements in addition to ECGP (ECGP + RTE + SUP).

### 2.4. Statistical Analysis

Statistical analyses were conducted in R, version 3.6.1 (R Foundation, Vienna, Austria), using survey package, version 4.0. Analyses of demographic information, blood folate concentrations, and folic acid intake group used corresponding NHANES sample weights to account for probabilistic selection and nonparticipation. An 8-year combined dietary recall weight was calculated from available 2-year dietary recall weights in NHANES 2011–2016. Due to differences in sampling procedures for NHANES 2017–2018, a 2-year folate-dietary subsample weight was derived from the 2-year dietary recall weight for 2017–2018 to account for the probabilistic selection and nonparticipation of available folate biomarker data. The combined 8-year weight was used to calculate adjusted geometric means, frequencies, and chi-squared and Wald tests (including Wald test for the CKD risk coefficient in fitted linear models).

## 3. Results

A total of 18,125 people ≥16 with available kidney biomarker data and dietary recall data were available for analysis; by CKD risk group there were 15,238 people considered low risk, 2070 with moderate risk, 562 with high risk, and 255 with highest risk (Table 1). 

### 3.1. Demographic Characteristics

Between CKD risk groups, there were statistically significant differences in age, sex, race/Hispanic origin, education, PIR, and BMI (*p* < 0.001 in all cases; Table 1). There was no statistically significant difference in folic acid intake group and CKD risk group (*p* = 0.16).

### 3.2. Folic Acid Usual Intake

No differences were found in modeled usual intake (median and IQR) of folic acid, natural food folates, total folates (sum of natural food folates and folic acid, in DFE), or total folic acid (total folates, converted to µg) when stratified by age (16–59, 60–74, ≥75) or by racial/ethnic groups (*p* > 0.05 in all cases; Table 2). Differences in modeled usual intake of folic acid, natural food folates, total folates, and total folic acid were found by folic acid intake sources (*p* < 0.0001 in all cases). Modeled usual folic acid intakes were similar across CKD risk groups (low risk: 226 (IQR: 110, 389) µg/day; moderate risk: 212 (IQR: 97, 381) µg/day; high risk: 257 (IQR: 112, 459) µg/day; highest risk: 234 (IQR: 103, 419) µg/day; *p* = 0.61). Natural food folate intakes were also similar across all CKD risk groups (Low Risk: 206 (IQR: 150, 270) dietary folate equivalent (DFE)/day; moderate risk: 189 (IQR: 139, 246) DFE/day; high risk: 176 (IQR: 132, 225) DFE/day; Highest Risk: 150 (IQR: 108, 198) DFE/day; *p* = 0.93). Finally, the modeled usual intakes of total folate, calculated as the sum of natural food folates and folic acid (in DFE), and total folic acid (total folates (DFE) converted to µg), were similar across all CKD risk groups (*p* = 0.72).

### 3.3. RBC Folate Concentrations

**Figure 1:**
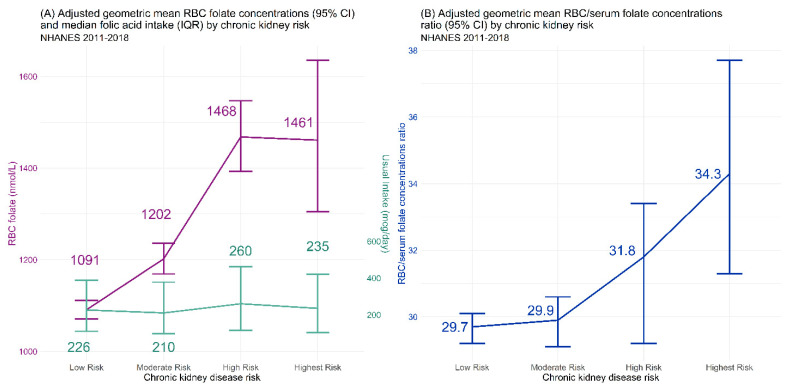
Red blood cell (RBC) folate concentrations, modeled folic acid usual intake, and RBC-to-serum total folate concentrations ratio by chronic kidney disease (CKD) risk group. CKD risk determined by eGFR and albuminuria stages as outlined by the Kidney Disease Improving Global Outcomes workgroup.

**Figure 2:**
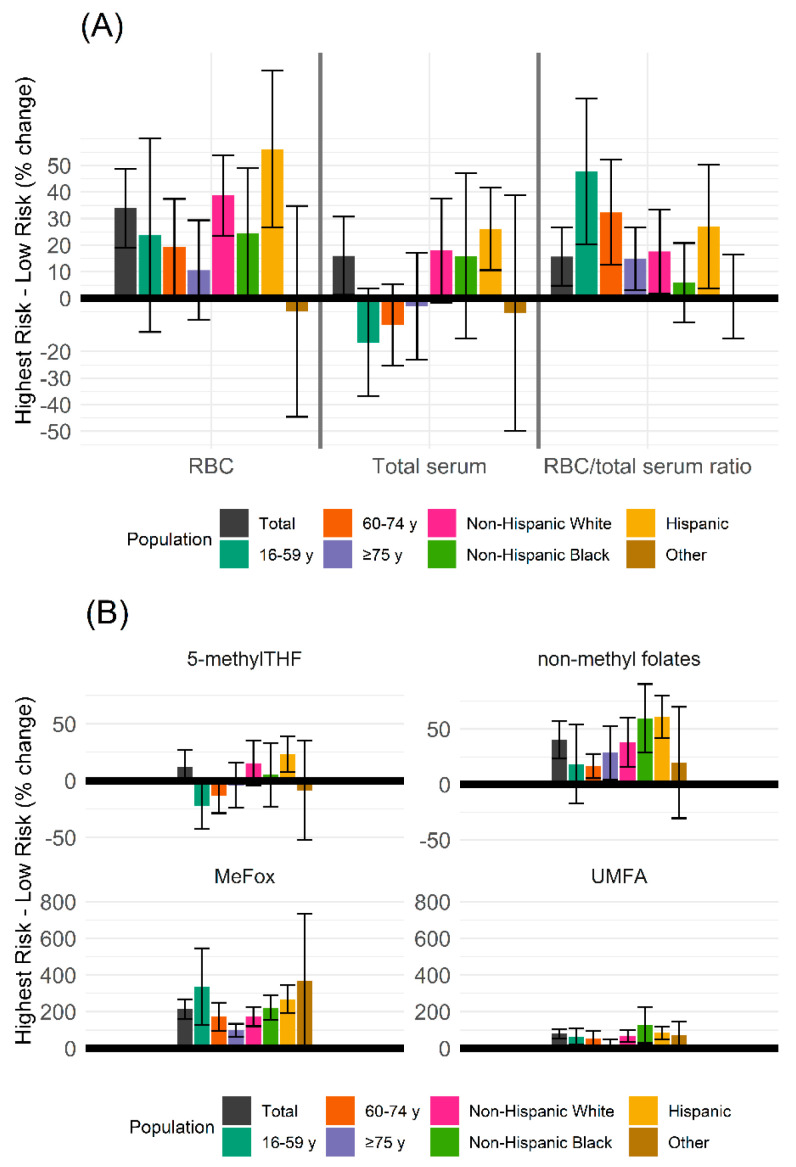
Percentage change in red blood cell (RBC) and serum folate concentrations, total and individual forms, between highest chronic kidney disease (CKD) risk and the low CKD Risk. Percent change of folate forms between the highest CKD Risk group and the low risk group (%), error bars represent the 95% CI for percentage change. CKD risk determined by eGFR and albuminuria stages as outlined by the Kidney Disease Improving Global Outcomes workgroup. Positive numbers indicate higher concentrations in the highest CKD risk group when compared to low risk group. The shaded gray areas represent the estimated percentage change in the overall population. Percentage change stratification by age and race/Hispanic origin are overlayed in color. (**A**) Percentage change in red blood cell (RBC) and serum total folate concentrations and RBC/serum ratio. Serum total folate is the sum of folate forms (5-methylTHF, non-methyl folate, folic acid) excluding MeFox. (**B**) Percentage change in individual serum folate forms. Non-methyl folate is the sum of 3 minor forms: THF, 5-formylTHF, and 5,10-methenylTHF. 5-methylTHF and non-methyl folates share the same *y*-axis scale, MeFox and UMFA share a separate *y*-axis scale.

The aGM of RBC folate concentrations showed a stepwise and significant (*p* < 0.0001; Table 3) increase as kidney function declined: the aGM among the low CKD risk group was 1089 nmol/L (95% CI: 1069, 1110 nmol/L), the moderate CKD risk group had higher RBC folate concentrations at 1189 nmol/L (95% CI: 1158, 1220 nmol/L), increasing higher among the high CKD risk group at 1488 nmol/L (95% CI: 1419, 1561 nmol/L), which was similar to the highest CKD risk group with 1443 nmol/L (95% CI: 1302, 1598 nmol/L). While RBC folate concentrations increased stepwise, it was independent of modeled folic acid usual intake, which remained similar at each CKD risk group (Figure 1A). The overall increase between the low and highest CKD risk was 33.4% (95% CI: 19.4%, 45.5%; Figure 2A).

The stepwise increase of RBC folate concentration aGM as kidney function declined was present regardless of age group (16–59: *p* = 0.0008; 60–74: *p* = 0.0072; ≥75: *p* = 0.0008; Table 3) or race/Hispanic origin group (Hispanic: *p* < 0.0001; non-Hispanic White: *p* < 0.0001; non-Hispanic Black: *p* < 0.0001; other: *p =* 0.022).

### 3.4. Serum Total Folate and Folate Forms

Overall, there were differences in the aGM of serum total folate concentrations by CKD risk group (low risk: 37.1 (95% CI: 36.3, 38.0) nmol/L; moderate risk: 40.2 (38.8, 41.7) nmol/L; high risk: 48.0 (44.3, 52.1) nmol/L; highest risk: 42.8 (95% CI: 37.8, 48.4) nmol/L; *p* < 0.0001; Table 3). The percentage increase between low and highest risk was 15.2% (95% CI: 1.0%, 29.4%; Figure 2A). When stratified by race/Hispanic origin, differences were found among Hispanic (*p* < 0.0001), non-Hispanic White groups (*p* < 0.0001), and non-Hispanic Black (*p* = 0.022), but differences were not significant among other (*p* = 0.06). When analyzed by age group, no statistical differences were found in serum total folate concentrations by CKD risk group (16–59: *p* = 0.77; 60–74: *p* = 0.066; ≥75: *p* = 0.18).

Serum 5-methylTHF showed a similar pattern as serum total folate, having overall significant differences stratified by CKD risk group (*p* < 0.0001; Table 3) with a 11.4% (95% CI: −2.8%, 25.5%; Figure 2B) difference between low and highest risk group, among Hispanic (*p* < 0.0001), non-Hispanic White (*p* < 0.0001), people aged 60–74 (*p* = 0.02). Differences were not found among non-Hispanic Black (*p* = 0.071), Other Hispanic origin (*p* = 0.11), people aged 16–59 (*p* = 0.62), and people aged ≥75 (*p* = 0.20).

Serum UMFA concentrations had a stepwise increase as kidney function declined (Low Risk: 0.81 (95% CI: 0.78, 0.84) nmol/L; Moderate Risk: 0.89 (95% CI: 0.84, 0.94) nmol/L; High Risk: 1.22 (95% CI: 1.09, 1.37) nmol/L; Highest Risk: 1.46 (95% CI: 1.26–1.68) nmol/L; *p* < 0.0001; Table 3). Between the low and highest risk, the increase was 80.2% (95% CI: 52.8%, 108%; Figure 2B). The increase was consistent among people 60–74 (*p* = 0.02) and those ≥75 (*p* = 0.023), though not reaching statistical significance among people 16–59 (*p* = 0.90). Differences were found among all Hispanic origin groups (*p* = 0.036 for other, *p* < 0.0001 in all other cases).

Serum non-methyl folate concentrations demonstrated mild increases as kidney function declined (low risk: 1.2 (95% CI: 1.1, 1.2) nmol/L; moderate risk: 1.2 (95% CI: 1.2, 1.3) nmol/L; high risk: 1.5 (95% CI: 1.4, 1.6) nmol/L; highest risk: 1.6 (95% CI: 1.4, 1.8) nmol/L; *p* < 0.0001; Table 3), with a relative increase from low to highest risk of 48.9% (95% CI: 21.5, 56.3; Figure 2B). The increase was seen across all age groups, though not reaching statistical significance among people 60–74 (16–59: *p* = 0.024; 60–74: *p* = 0.13; ≥75: *p* = 0.0006). The progressive increase was significant among all Hispanic origin groups (*p* = 0.0097 for other, *p* < 0.0001 in all other cases).

Finally, MeFox demonstrated a robust increase as CKD risk increased: low risk began at 1.4 nmol/L (95% CI: 1.4, 1.5 nmol/L; increased to 1.8 nmol/L (95% CI: 1.7, 1.8 nmol/L) in moderate risk, 2.7 nmol/L (95% CI: 2.5, 2.9 nmol/L) in the high risk group, and to 4.4 nmol/L (95% CI: 3.8, 5.0 nmol/L) in the highest risk group (*p* < 0.0001) (Table 3), a 202% (95% CI: 162%, 243%) increase from low to highest risk (Figure 2B). This increase was consistent across all age and Hispanic origin groups (*p* < 0.0001 in all cases).

### 3.5. RBC/Serum Ratio

A ratio of RBC folate concentration to serum total folate concentration controls for recent folate intake. Overall, the RBC/serum total folate ratio progressively increased as kidney function declined (low risk: 29.3 (95% CI: 28.9, 29.7); moderate risk: 29.6 (95%CI: 28.8, 30.3); High Risk: 31.0 (29.0, 33.2); highest risk: 33.8 (95% CI: 30.8, 37.1); *p* = 0.046; Figure 1B; Table 3). This increase in RBC/serum total folate ratio existed across age groups (16–59: *p* = 0.0012; 60–74: *p* < 0.0001; ≥75: *p* = 0.028). When stratified by race/Hispanic origin, the progressive increase was significant among non-Hispanic White (*p* = 0.042) and non-Hispanic Black (*p* = 0.02) populations, but not among Hispanic (*p* = 0.13) and other (*p* = 0.85). 

### 3.6. Liver Measures

ALT aGM concentrations showed differences by CKD risk group (low risk: 22.2 (95% CI: 22.0, 22.5) international units (IU)/L; moderate risk: 21.5 (95% CI: 20.9, 22.0) IU/L; high risk: 20.5 (95% CI: 19.6, 21.4) IU/L; highest risk: 18.2 (95% CI: 16.7, 19.9) IU/L; *p* = 0.0002). AST aGM concentrations showed small differences by CKD risk group that were significant (low risk: 23.9 (95% CI: 23.7, 24.1) IU/L; moderate risk: 24.7 (95% CI: 24.2, 25.2) IU/L; high risk: 25.0 (95% CI: 24.1, 25.9) IU/L; highest risk: 22.1 (20.5, 23.8) IU/L; *p* = 0.054).

## 4. Discussion

The current analyses explore the relationships between kidney function (measured CKD risk), usual folic acid intake, and RBC and serum folate concentrations using NHANES 2011–2018 data. There was no association between folic acid intake source and CKD risk and no association between folic acid usual intake and CKD risk, indicating that all differences in folate concentrations across CKD risk groups were independent of usual folic acid intake. RBC folate concentrations increased with declining kidney function, suggesting homeostatic changes in kidney function have an impact on RBC folate concentrations independent of dietary folic acid intake. The relationship between serum total folate and level of kidney function differed by age and race/Hispanic origin categories.

### 4.1. RBC and Serum Total Folates

Serum total folate concentration is often considered an indicator of recent folate intake, whereas RBC folate concentration is considered an indicator of long-term folate status [17]. Overall, we found increases between low and highest CKD risk group in both RBC and serum folate concentrations (Figure 2A). Because both RBC folate and serum folate concentrations are on the same scale (nmol/L), the greater increase in RBC folate than in serum folate concentrations represents a greater flux in folate status. The RBC folate concentration to serum total folate concentration ratio, which controls for intra-individual variance in recent folic acid intake, also increased as CKD risk increased. The increase in the ratio held across all age and race/Hispanic origin groups, except for other race, which had smaller sample sizes for high and highest CKD risk groups. Taken together, the increase in RBC folate concentrations and the increase in the RBC-to-serum folate concentration ratio suggest that metabolic and homeostatic changes that occur due to changes in kidney function can lead to elevated RBC folate concentration that is independent of usual intake, recent folate intake, and intra-individual variance.

### 4.2. Serum Folate Forms

There was an overall increase in serum 5-methylTHF concentrations between low and highest CKD risk groups (Figure 2B). Stratified by age group, our analyses showed that differences may be driven by age, reaching significance in people 60–74, suggesting a potential interaction of age and kidney function on 5-methylTHF concentration. Making up approximately 95% of all serum folate forms, 5-methylTHF is also the primary folate form reabsorbed by the kidneys [2]. The increase in folate concentrations can be due to several underlying biological processes: increases may potentially be due to a methyl trap wherein, under potential low methionine conditions due to CKD risk, folate becomes trapped as 5-methylTHF. CKD also reduces albumin, a plasma carrier protein that approximately 50% of plasma folates are bound to, and as more albumin is lost, less is available for folate transfer and metabolic turnover resulting in increased folate concentrations. Finally, reduced kidney function potentially increases exposure to toxicants, and an upregulation of folate transport to aid in detoxification may lead to increased folate concentrations. These initial results suggest a complex interaction between kidney function, folate metabolism, and reabsorption to be explored further.

Serum MeFox concentrations showed the most robust increase as kidney function declined (Figure 2B). MeFox is an oxidation product of 5-methylTHF. While it is possible that MeFox can be generated post-blood collection, it is hypothesized to be already present in vivo and may provide insights into folate metabolism [18,19]. In this framework, analyses of MeFox have been closely tied to several negative health factors, including obesity, smoking, inflammation, and low eGFR [19]. Our findings are consistent with these previous studies, and crucially this analysis demonstrates that the increase in serum MeFox concentrations is independent of folic acid usual intake, which remained the same across CKD risk groups.

Serum UMFA concentrations increased from low and highest CKD risk groups (Figure 2B). This relationship held true across age and race/Hispanic origin. Serum UMFA concentrations have been correlated with folic acid dietary intake and supplementation and it has been suggested that UMFA may be an indicator of a mismatch between folic acid intake and cellular demand for folic acid [19,20]. Our results indicate this relationship is more complex than previously suggested. In addition, these data suggest that as kidney function decreases, there is less export of excess folic acid resulting in increase in UMFA although our reported changes are very small on the absolute scale of a half nmol/L increase.

Serum non-methyl folates increased with decreasing kidney function from Low to Highest Risk groups (Figure 2B). One of the primary folate forms in non-methyl folates is THF, which is produced early in UMFA reduction. While THF concentration has been tied to UMFA concentrations, increases in non-methyl folates appear to be rate-limited [19]. Indeed, the percentage change in non-methyl folate was about half of the change seen in serum UMFA concentrations.

### 4.3. Liver Biomarkers

We further analyzed biomarkers of liver functioning to provide a more complete picture of metabolic and homeostatic changes that might occur with changes in kidney function. ALT and AST levels differed significantly by CKD risk group (*p* = 0.0002, *p* = 0.054 respectively; data not shown). The trends between liver biomarkers and CKD risk were unclear and measures appeared to be within the normal range across all CKD risk groups. While there may be an interaction between kidney function and liver health, the current analyses suggest reduced kidney function is the main contributor to elevating RBC folate concentrations independent of liver function.

### 4.4. Implications for Researchers

These data show a complex interaction between kidney function, usual folic acid intake, and RBC and serum folate concentrations. These interactions are apparent in a nationally representative population-based study, indicating a relationship that occurs early during disease progression and likely prior to clinical CKD diagnosis. Importantly, our analyses demonstrate elevated RBC folate concentrations can be indicative of changes in metabolic and homeostatic processes independent of folic acid usual intake. Scientific studies continue to explore potential adverse impacts of high intakes or blood concentrations of folates, and specifically folic acid, with inconsistent results. Many of these studies use folate concentrations as a proxy for folic acid intake, assuming that correlations between potential adverse impacts and high RBC folates are due largely to high folic acid intake (e.g., kidney failure in pre-eclampsia and fetal outcomes). Our analyses suggest this use of folate concentrations as a proxy for folic acid intake fails to consider a complex system of homeostatic and metabolic processes that contribute to RBC folate concentrations.

**Figure 3:**
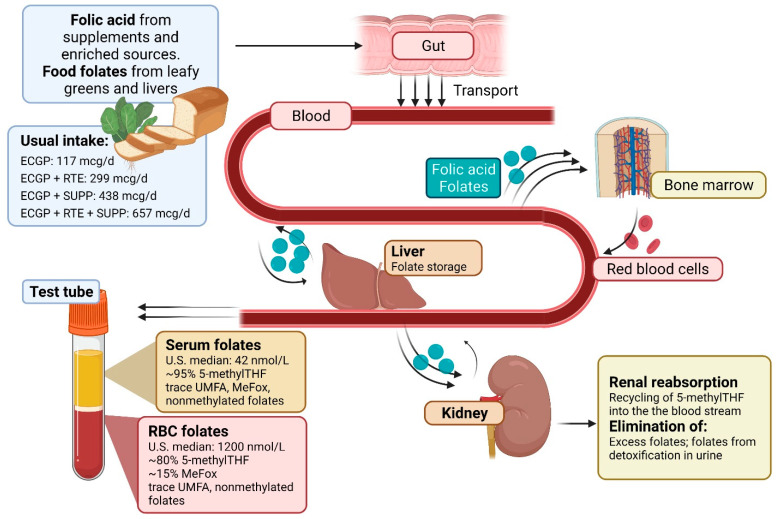
Pathway for folate metabolism. Diagram depicting the metabolism of folic acid and food folates; ECGP: enriched cereal grain product; ECGP + RTE: enriched cereal grain product plus ready-to-eat cereals; ECGP + SUPP: enriched cereal grain product plus folic acid containing supplements; ECGP + RTE + SUPP: enriched cereal grain product plus ready-to eat cereals plus folic acid containing supplements. Folic acid and food folates are transported into the blood stream via the small intestine. The liver then aids in the biotransformation of food folates to 5-methylTHF where it is either stored via polyglutamylation or re-enters the bloodstream where it is circulated to peripheral tissues, including bone marrow where it is actively transported into red blood cells. Circulating blood folates are filtered in the kidney, where a portion is reabsorbed, and excess and reduced forms are excreted in the urine [19,24]. Created with BioRender.com (accessed on 26 January 2022).

Folate concentrations taken from RBC or serum are not a direct measure of intake, but rather a product of biological processes that occur between intake and measurement. As illustrated in Figure 3, both folic acid and food folates are absorbed primarily from the small intestines via protein proton-coupled folate receptors. Once absorbed, folate exists freely in serum [21]. Absorbed folates in serum are then transferred to the liver, where some folic acid and folates are removed. Removed folic acid and folates undergo biotransformation at the liver to 5-methylTHF and are then partially released into bile which allows for reabsorption from the small intestine [22]. Converted 5-methylTHF in the liver is either stored via polyglutamylation or it enters the hepatic vein where it is circulated to meet the one-carbon requirements of peripheral tissues, including bone marrow tissue where it is actively transported into red blood cells. Circulating blood folates that are not bound to serum proteins are filtered in the kidney, where folate receptor α (FRα) is highly expressed along tubule epithelial cells. FRα has a high binding affinity to 5-methylTHF and folic acid, allowing for a highly efficient reabsorption mechanism of biologically available folate forms [23]. Excess folates after reabsorption and reduced folate forms are then eliminated in the urine by the kidneys. RBC and serum folate concentrations are not just a product of dietary intake; they reflect all metabolic processing including intestinal absorption, liver processes, and reabsorption by the kidneys.

Generally, RBC folates are thought of as a more stable biomarker whereas serum folates are more variable and reflects recent folate intakes, although it has been suggested that RBC folate concentrations are mediated by vitamin B_12_ [25]. RBC and serum folate concentrations are measured on the same scale (nmol/L) and generally correlate with one another [26]. When the correlation between RBC and serum folate concentrations begins to diverge, it is likely reflective of changes in the underlying biological processes in folate metabolism and homeostasis. Tracking this ratio might be an important indicator of when metabolism is going out of homeostasis.

Current analyses demonstrate that reductions in kidney function, as measured by CKD risk, have a notable impact on RBC and serum folate concentrations. It is highly recommended to use caution when interpreting studies that associate high folate concentrations with adverse outcomes, specifically if they are limited to a small sample size as these associations may reflect reduced kidney function.

### 4.5. Limitations and Considerations

NHANES data are cross-sectional in nature, so we cannot directly assess the causal relationship between kidney function and RBC or serum folate concentrations. Additionally, CKD risk is typically determined with multiple measurements taken over several months, whereas NHANES data are limited to measurements at a single timepoint. Previous analyses of follow-up data have shown that having only one measurement can lead to an overestimation of albuminuria stage [27,28]. We conducted additional analyses stratifying the population by either eGFR or albuminuria stages, rather than the combined CKD risk groups; examining albuminuria or eGFR stages independently found similar increases in folate concentrations (see Supplemental Table S1). Additional consideration was made regarding the inclusion of a racial adjustment in calculating eGFR [29]. We found that neither removing the race adjustment from the standard eGFR equation and adjusting for race/Hispanic origin in the model nor including sex and non-Hispanic origin adjustments in our model with the standard eGFR equation affected our findings (see Supplemental Tables S2 and S3). It is important to note that most individuals in the U.S. with CKD are unaware of their condition or its severity; thus, it is crucial for medical providers to use available biomarkers to diagnose CKD [30].

Finally, approximately 1 in 13 Blacks (African Americans) is born with the sickle cell trait [31], which is associated with several renal complications including hematuria, isosthenuria and renal medullary carcinoma and has been demonstrated to increase CKD risk [32,33]. The impact of the sickle-cell trait on circulating folates via kidney function is an area in need of additional research. Unfortunately, NHANES does not provide information on sickle-cell trait, limiting our ability to control for it in our analyses.

## 5. Conclusions

The ongoing study of adverse impacts of high folic acid intake or high folate concentration remains inconsistent in the literature. These studies rely largely on the correlation between high intake and folate concentrations. At the population level, there might be a reasonable correlation between folic acid intake and folate biomarkers. However, our current analyses have shown that the correlation between intake and RBC folate concentrations can be mediated by kidney function. When looking for associations of disease outcomes, researchers may want to consider the mediating role of kidney function in the relationship between folic acid intake and folate biomarkers, which in turn may confound associations between folic acid concentrations and disease outcome.

## Figures and Tables

**Table 1:** Demographic characteristics of US individuals ≥16 stratified by chronic kidney disease (CKD) risk, NHANES 2011–2018 ^1^.

	Low Risk		Moderate Risk		High Risk		Highest Risk		*p* Value ^2^
	*n*	Weighted % (95% CI)	*n*	Weighted % (95% CI)	*n*	Weighted % (95% CI)	*n*	Weighted % (95% CI)	
Total	15,238		2070		562		255		
Age									
16–59	11,855	80.6 (79.2, 82.0)	982	41.4 (48.5, 54.3)	156	33.2 (27.1, 39.3)	47	23.2 (15.1, 32.3)	<0.0001
60–74	2710	15.8 (14.5, 17.0)	665	30.4 (27.6, 33.2)	175	25.5 (20.3, 30.8)	82	27.3 (19.9, 34.7)	
≥75	673	3.7 (3.3, 4.1)	423	18.2 (16.2, 20.2)	231	41.3 (36.6, 46.0)	126	49.5 (41.3, 57.7)	
Sex									0.0003
Male	7296	48.1 (47.1, 49.22)	918	40.8 (37.4, 44.2)	271	42.4 (35.9, 48.8)	147	48.0 (40.9, 55.1)	
Female	7942	51.9 (50.8, 52.9)	1152	59.2 (55.8, 62.6)	291	57.6 (51.2, 64.1)	108	52.0 (44.8, 59.1)	
Race/Hispanic origin									<0.0001
Non-Hispanic White	5529	63.7 (59.9, 67.4)	875	66.9 (62.4, 71.3)	273	71.3 (66.4, 76.2)	108	63.2 (54.1, 72.3)	
Non-Hispanic Black	3313	10.9 (8.9, 12.9)	488	12.1 (9.2, 14.9)	130	12.5 (9.3, 15.6)	67	15.8 (10.1, 21.6)	
Hispanic	3931	16.2 (13.6, 18.8)	464	13.0 (9.9, 16.0)	102	9.6 (6.7, 12.5)	52	11.2 (6.7, 15.7)	
Other	2465	9.3 (8.0, 10.5)	243	8.1 (6.3, 9.9)	57	6.7 (4.4, 8.9)	28	9.8 (5.1, 14.5)	
Education									<0.0001
<High School	3819	16.6 (14.9, 18.3)	618	20.9 (18.4, 23.3)	157	21.9 (18.1, 25.7)	80	24.3 (17.6, 31.0)	
High School graduate/GED	3288	21.6 (20.1, 23.2)	478	23.3 (20.4, 26.2)	134	22.0 (17.6, 26.4)	71	32.1 (23.8, 40.4)	
>High School	8104	61.6 (59.1, 64.1)	971	55.8 (52.0, 59.6)	270	56.1 (50.8, 61.4)	104	43.6 (36.3, 50.9)	
Missing	27	—	3	—	1	—	0	—	
Poverty/Income Ratio									0.0001
<1.0	3153	14.7 (12.9, 16.4)	461	16.9 (13.8, 18.2)	125	17.2 (11.3, 23.1)	59	23.7 (15.9, 31.5)	
1.0—1.9	3384	17.6 (16.2, 18.9)	529	20.9 (18.3, 23.5)	158	24.1 (18.3, 30.0)	71	24.3 (19.0, 29.6)	
2.0—3.9	3507	24.9 (23.0, 26.7)	468	25.6 (22.6, 28.6)	120	23.7 (19.6, 27.8)	65	25.1 (18.6, 31.6)	
≥4.0	3545	33.3 (30.6, 36.1)	384	28.7 (24.7, 32.7)	91	25.1 (19.3, 30.9)	28	—	
Missing	1649	—	228	—	68	—	32	—	
BMI									<0.0001
Underweight (BMI < 18.5)	310	1.7 (1.4, 2.0)	55	2.5 (1.6, 3.4)	12	—	3	—	
Normal Weight (18.5 ≤ BMI < 25)	4665	30.0 (28.6, 32.4)	506	24.6 (22.2, 27.0)	103	17.5 (14.0, 21.0)	54	19.1 (13.2, 25.1)	
Overweight (25 ≤ BMI < 30)	4735	31.8 (30.5, 33.1)	591	28.1 (25.0, 31.2)	176	32.3 (26.6, 38.0)	80	26.6 (19.5, 33.8)	
Obese (BMI ≥ 30)	5424	36.0 (34.5, 37.5)	894	43.8 (40.7, 46.9)	257	46.2 (40.2, 52.1)	110	50.9 (41.3, 60.5)	
Missing	104	—	24	—	14	—	8	—	
Folic Acid source									0.23
ECGP only	8355	51.2 (49.5, 52.9)	1100	49.2 (45.6, 52.7)	270	42.4 (36.7, 48.0)	133	49.0 (39.8, 58.1)	
ECGP + SUP	3049	21.9 (20.7, 23.1)	419	22.4 (19.5, 25.4)	125	25.9 (20.4, 31.6)	52	27.7 (17.2, 38.3)	
ECGP + RTE	2690	17.5 (16.7, 18.3)	380	19.0 (16.2, 21.8)	110	20.4 (15.6, 25.2)	49	16.0 (10.2, 21.9)	
ECGP + RTE + SUP	1138	9.3 (8.4, 10.3)	171	9.4 (7.6, 11.1)	57	11.3 (8.0, 14.6)	21	—	
Missing	6	—	0	—	0	—	0	—	

^1^ Values represent the weighted proportion of the population (95% CI) within each CKD risk group; CKD risk determined by eGFR and albuminuria stages as outlined by the Kidney Disease Improving Global Outcomes workgroup; estimated proportions for sample sizes <30 have been suppressed; ECGP, enriched cereal grain product; ECGP + SUP, enriched cereal grain product plus folic acid containing supplements; ECGP + RTE, enriched cereal grain product plus ready-to-eat cereals; ECGP + RTE + SUP, enriched cereal grain product plus ready-to-eat cereals plus folic acid containing supplements. ^2^ *p* values calculated from the χ^2^ test.

**Table 2:** Modeled usual intake of folic acid and total folates, in demographic subgroups ≥16 y, NHANES 2011–2018 ^1^.

	*n*	Folic Acid (µg)	Natural Food Folate (DFE)	Total Folate (DFE) ^2^	Total Folic Acid (µg) ^3^
Total	18,127	225 (108, 390)	201 (146, 264)	610 (372, 914)	366 (223, 548)
Age					
16–59 y	13,041	219 (112, 369)	206 (149, 270)	603 (381, 884)	362 (229, 530)
60–74 y	3633	243 (101, 450)	202 (149, 264)	648 (369, 1014)	389 (221, 606)
≥75 y	1453	268 (111, 481)	172 (129, 219)	647 (362, 1013)	388 (217, 608)
*p* value ^4^		0.27	0.063	0.14	0.14
Race/Hispanic origin					
Hispanic	4550	190 (93, 318)	209 (146, 281)	555 (342, 810)	333 (205, 486)
Non-Hispanic White	6785	244 (120, 423)	200 (142, 258)	649 (403, 969)	389 (242, 581)
Non-Hispanic Black	3999	179 (86, 308)	168 (120, 220)	489 (291, 736)	293 (175, 442)
Other	2793	222 (108, 374)	216 (156, 286)	624 (391, 907)	374 (235, 544)
*p* value		0.38	0.88	0.59	0.59
Folic acid source					
ECGP only	9864	116 (78, 160)	194 (140, 255)	404 (305, 511)	242 (183, 307)
ECGP + RTE	3229	315 (276, 625)	202 (147, 266)	746 (625, 877)	448 (375, 526)
ECGP + SUP	3647	434 (272, 625)	216 (161, 280)	970 (688, 1299)	582 (413, 779)
ECGP + RTE + SUP	1387	653 (488, 839)	220 (167, 283)	1340 (1043, 1675)	804 (626, 1005)
*p* value		<0.0001	<0.0001	<0.0001	<0.0001
CKD risk					
Low Risk	15,238	226 (110, 389)	206 (150, 270)	617 (381, 917)	370 (229, 550)
Moderate Risk	2070	212 (97, 381)	189 (139, 246)	577 (349, 880)	346 (209, 528)
High Risk	563	257 (112, 459)	176 (132, 225)	644 (363, 999)	386 (218, 599)
Highest Risk	250	234 (103, 419)	150 (108, 198)	558 (307, 893)	335 (184, 535)
*p* value		0.61	0.93	0.72	0.72

^1^ Values represent the median usual intake and interquartile range (IQR) within each subpopulation; CKD risk determined by eGFR and albuminuria stages as outlined by the Kidney Disease Improving Global Outcomes workgroup; ECGP, enriched cereal grain product; ECGP + SUP, enriched cereal grain product plus folic acid containing supplements; ECGP + RTE, enriched cereal grain product plus ready-to-eat cereals; ECGP + RTE + SUP, enriched cereal grain product plus ready-to-eat cereals plus folic acid containing supplements; DFE, dietary folate equivalents. ^2^ Total folate (DFE) = natural food folate (DFE) + (folic acid (µg) × 1.7). ^3^ Total folate DFE was converted to total in µg folic acid; total in µg folic acid = total folate (DFE) × 0.6. ^4^ *p* values calculated from Wald test across each stratified subgroup.

**Table 3:** Concentrations of red blood cell (RBC) folate, serum total folate, and major folate forms in demographic subgroups ≥16, stratified by chronic kidney disease (CKD) risk group, NHANES 2011–2018 ^1^.

	CKD Risk Group				
Analyte	Low Risk	Moderate Risk	High Risk	Highest Risk	*p* Value ^2^
RBC folate (nmol/L)					
Overall	1089 (1069, 1110)	1189 (1158, 1220)	1488 (1419, 1561)	1443 (1302, 1598)	<0.0001
16–59	1053 (1032, 1074)	1075 (1035, 1117)	1277 (1169, 1394)	1345 (1084, 1669)	0.0008
60–74	1231 (1195, 1267)	1263 (1202, 1329)	1432 (1328, 1543)	1377 (1212, 1563)	0.0072
≥75	1387 (1330, 1446)	1445 (1365, 1529)	1736 (1621, 1859)	1512 (1285, 1780)	0.0008
Hispanic	998 (983, 1013)	1097 (1047, 1149)	1092 (1013, 1176)	1460 (1186, 1799)	<0.0001
Non-Hispanic White	1161 (1139, 1184)	1243 (1202, 1286)	1645 (1555, 1739)	1578 (1421, 1752)	<0.0001
Non-Hispanic Black	866 (847, 886)	973 (919, 1029)	1127 (1021, 1244)	1081 (894, 1307)	<0.0001
Other	1049 (1026, 1072)	1193 (1121, 1268)	1173 (1056, 1304)	1079 (792, 1469)	0.022
Serum folate (nmol/L) ^3^					
Overall	37.1 (36.3, 38.0)	40.2 (38.8, 41.7)	48.0 (44.3, 52.1)	42.8 (37.8, 48.4)	<0.0001
16–59	35.3 (34.5, 36.1)	35.7 (33.8, 37.8)	34.7 (32.0, 37.6)	30.4 (24.1, 38.2)	0.77
60–74	44.3 (42.3, 46.4)	41.3 (39.0, 43.8)	44.7 (39.1, 51.0)	38.0 (32.5, 44.4)	0.066
≥75	55.3 (52.3, 58.6)	54.5 (50.2, 59.2)	66.9 (59.4, 75.4)	53.6 (45.2, 63.5)	0.18
Hispanic	34.9 (34.2, 35.7)	38.2 (36.2, 40.3)	38.0 (33.5, 43.2)	44.5 (38.8, 51.1)	<0.0001
Non-Hispanic White	39.1 (38.1, 40.2)	41.9 (40.1, 43.8)	52.7 (47.5, 58.4)	45.1 (38.1, 53.5)	<0.0001
Non-Hispanic Black	29.6 (28.7, 30.4)	31.4 (29.5, 33.5)	34.3 (30.1, 39.0)	34.8 (27.1, 44.7)	0.022
Other	37.0 (35.8, 38.3)	42.8 (39.4, 46.6)	42.7 (35.7, 51.1)	36.5 (25.1, 53.2)	0.06
5-methylTHF (nmol/L)					
Overall	34.5 (33.7, 35.3)	37.3 (36.0, 38.7)	44.0 (40.5, 47.7)	38.4 (33.8, 43.7)	<0.0001
16–59	32.8 (32.0, 33.5)	33.3 (31.4, 35.3)	32.0 (29.2, 35.0)	26.4 (20.4, 34.1)	0.62
60–74	41.2 (39.3, 43.2)	38.4 (36.2, 40.7)	40.4 (35.9, 45.4)	34.0 (28.9, 40.0)	0.02
≥75	51.0 (48.3, 53.9)	50.1 (46.1, 54.4)	61.4 (54.4, 69.3)	49.0 (41.4, 58.0)	0.20
Hispanic	32.6 (31.8, 33.4)	35.7 (33.8, 37.8)	35.0 (30.3, 40.5)	40.5 (35.2, 46.6)	<0.0001
Non-Hispanic White	36.4 (35.4, 37.3)	39.0 (37.3, 40.7)	48.2 (43.4, 53.5)	41.1 (34.5, 48.9)	<0.0001
Non-Hispanic Black	26.9 (26.2, 27.7)	28.6 (26.7, 30.6)	31.0 (27.2, 35.4)	28.7 (22.3, 37.0)	0.071
Other	34.7 (33.5, 35.9)	39.8 (36.6, 43.3)	39.6 (33.4, 47.0)	33.2 (22.6, 48.6)	0.11
UMFA (nmol/L)					
Overall	0.81 (0.78, 0.84)	0.89 (0.84, 0.94)	1.22 (1.09, 1.37)	1.46 (1.26, 1.68)	<0.0001
16–59	0.77 (0.74, 0.79)	0.73 (0.69, 0.78)	0.74 (0.62, 0.88)	1.25 (0.94, 1.66)	0.90
60–74	0.95 (0.89, 1.02)	0.97 (0.87, 1.08)	1.24 (0.96, 1.62)	1.48 (1.12, 1.95)	0.02
≥75	1.34 (1.16, 1.54)	1.34 (1.15, 1.56)	1.87 (1.61, 2.18)	1.55 (1.30, 1.85)	0.023
Hispanic	0.68 (0.64, 0.71)	0.72 (0.66, 0.79)	0.78 (0.71, 0.86)	1.28 (1.02, 1.59)	<0.0001
Non-Hispanic White	0.87 (0.83, 0.90)	0.94 (0.87, 1.02)	1.37 (1.19, 1.58)	1.45 (1.20, 1.76)	<0.0001
Non-Hispanic Black	0.79 (0.76, 0.83)	0.87 (0.80, 0.95)	1.04 (0.89, 1.22)	1.79 (1.19, 2.70)	<0.0001
Other	0.67 (0.64, 0.71)	0.74 (0.60, 0.91)	0.81 (0.54, 1.22)	1.15 (0.74, 1.80)	0.036
Non-methyl folates (nmol/L) ^4^					
Overall	1.2 (1.1, 1.2)	1.2 (1.2, 1.3)	1.5 (1.4, 1.6)	1.6 (1.4, 1.8)	<0.0001
16–59	1.1 (1.1, 1.2)	1.1 (1.1, 1.2)	1.3 (1.1, 1.4)	1.3 (1.0, 1.6)	0.024
60–74	1.3 (1.2, 1.4)	1.3 (1.2, 1.4)	1.5 (1.3, 1.7)	1.4 (1.3, 1.6)	0.13
≥75	1.5 (1.4, 1.6)	1.6 (1.4, 1.7)	1.8 (1.6, 2.0)	1.9 (1.5, 2.3)	0.0006
Hispanic	1.1 (1.0, 1.3)	1.2 (1.1, 1.4)	1.4 (1.1, 1.8)	1.8 (1.6, 2.2)	<0.0001
Non-Hispanic White	1.2 (1.1, 1.2)	1.2 (1.2, 1.3)	1.6 (1.4, 1.7)	1.6 (1.3, 1.9)	<0.0001
Non-Hispanic Black	1.1 (1.0, 1.3)	1.3 (1.1, 1.4)	1.4 (1.2, 1.6)	1.8 (1.5, 2.2)	<0.0001
Other	1.1 (1.1, 1.2)	1.3 (1.1, 1.4)	1.3 (1.0, 1.6)	1.5 (1.1, 2.0)	0.0097
MeFox (nmol/L)					
Overall	1.4 (1.4, 1.5)	1.8 (1.7, 1.8)	2.7 (2.5, 2.9)	4.4 (3.8, 5.0)	<0.0001
16–59	1.4 (1.4, 1.4)	1.5 (1.4, 1.6)	1.9 (1.6, 2.3)	5.1 (3.8, 7.0)	<0.0001
60–74	1.6 (1.6, 1.7)	1.9 (1.8, 2.1)	2.8 (2.5, 3.2)	4.3 (3.2, 5.8)	<0.0001
≥75	1.9 (1.8, 2.1)	2.4 (2.2, 2.6)	3.4 (3.1, 3.7)	4.0 (3.4, 4.6)	<0.0001
Hispanic	1.2 (1.2, 1.3)	1.4 (1.3, 1.6)	1.9 (1.6, 2.2)	4.2 (3.3, 5.2)	<0.0001
Non-Hispanic White	1.6 (1.5, 1.6)	1.9 (1.8, 2.0)	3.0 (2.8, 3.2)	4.2 (3.6, 4.9)	<0.0001
Non-Hispanic Black	1.1 (1.1, 1.1)	1.4 (1.3, 1.5)	1.7 (1.3, 2.1)	3.7 (3.0, 4.4)	<0.0001
Other	1.5 (1.4, 1.5)	1.6 (1.4, 1.8)	2.3 (1.8, 2.9)	5.6 (3.4, 9.2)	<0.0001
RBC/serum ratio					
Overall	29.3 (28.9, 29.7)	29.6 (28.8, 30.3)	31.0 (29.0, 33.2)	33.8 (30.8, 37.1)	0.0046
16–59	29.9 (29.4, 30.3)	30.0 (28.8, 31.3)	36.8 (32.8, 41.3)	44.2 (37.1, 52.5)	0.0012
60–74	27.8 (26.9, 28.7)	30.6 (29.3, 31.9)	32.0 (28.7, 35.8)	36.2 (31.0, 42.4)	<0.0001
≥75	25.0 (24.1, 25.9)	26.5 (25.2, 27.9)	25.9 (24.1, 27.9)	28.4 (26.0, 31.1)	0.028
Hispanic	28.6 (28.0, 29.1)	28.7 (27.6, 29.9)	28.7 (26.0, 31.7)	33.8 (27.6, 41.5)	0.13
Non-Hispanic White	29.7 (29.1, 30.3)	29.7 (28.7, 30.6)	31.2 (28.6, 34.1)	35.0 (30.6, 40.0)	0.042
Non-Hispanic Black	29.3 (28.7, 30.0)	30.8 (29.4, 32.2)	32.9 (30.1, 35.9)	30.5 (26.8, 34.8)	0.02
Other	28.3 (27.6, 29.0)	27.8 (26.2, 29.6)	27.5 (23.7, 31.9)	29.5 (25.0, 34.8)	0.85

^1^ Values represent the adjusted geometric mean (95% CI), adjusting for body mass index, poverty-income ratio, and education levels; CKD risk determined by eGFR and albuminuria stages as outlined by the Kidney Disease Improving Global Outcomes workgroup; ^2^ *p* values calculated from trend test within demographic subgroup across CKD risk groups. ^3^ Serum total folate is the sum of folate forms (5-methylTHF, non-methyl folate, folic acid) excluding MeFox. ^4^ Non-methyl folate is the sum of 3 minor forms: THF, 5-formylTHF, and 5,10-methenylTHF; non-methyl folate is below level of detection (<LOD) if all 3 minor forms were <LOD.

## Data Availability

Publicly available datasets were analyzed in this study. The data can be found here: https://wwwn.cdc.gov/nchs/nhanes/default.aspx. Data last accessed 21 January 2022.

## Supplementary Materials

The following are available online at https://www.mdpi.com/article/10.3390/nu14051054/s1, Table S1: Concentrations of red blood cell (RBC) folate and serum total folate, RBC/serum ratio, and folic acid usual intake in persons aged ≥16, stratified by albuminuria stages, NHANES 2011–2018, Table S2: Concentrations of red blood cell (RBC) folate and serum total folate, RBC/serum ratio, and folic acid usual intake in persons aged >16, stratified by estimated glomerular filtration rate (eGFR) stages, NHANES 2011–2018, Table S3: Concentrations of red blood cell (RBC) folate and serum total folate and RBC/serum ratio in demographic subgroups aged >16, stratified by chronic kidney disease (CKD) risk group, with additional adjustments, NHANES 2011–2018, Figure S1: Participant flowchart of NHANES 2011–2018.

## Abbreviations

5,10-methenylTHF	5,10-methenyletrahydrofolate
5-formylTHF	5-formyltetrahydrofolate
5-methylTHF	5-methyltetrahydrofolate
ACR	Albumin-to-creatinine ratio
aGM	Adjusted geometric mean
ALT	Alanine aminotransferase
AST	aspartate aminotransferase
BMI	Body mass index
CKD	Chronic kidney disease
CKD-EPI	Chronic Kidney Disease Epidemiology Collaboration
DFE	Dietary folate equivalents
ECGP	Enriched cereal grain product
eGFR	Estimated glomerular filtration rate
GED	General Equivalency Diploma
KDIGO	Kidney Disease: Improving Global Outcomes
NHANES	National Health and Nutrition Examination Survey
MEC	Mobile examination center
NCI	National Cancer Institute
MeFox	Pyrazino-s-triazine derivative of 4-α-hydroxy-5-methyltetrahydrofolate
PIR	Poverty-income ratio
RBC	Red blood cell
RTE	Ready-to-eat cereals
SUP	Folic acid containing supplements
THF	Tetrahydrofolate
UMFA	Unmetabolized folic acid

## References

[1] National Toxicology Program (2015). NTP Monograph: Identifying Research Needs for Assessing Safe Use of High Intakes of Folic Acid.

[2] Samodelov S.L., Gai Z., Kullak-Ublick G.A., Visentin M. (2019). Renal Reabsorption of Folates: Pharmacological and Toxicological Snapshots. Nutrients.

[3] Lowrance W.T., Ordonez J., Udaltsova N., Russo P., Go A.S. (2014). CKD and the risk of incident cancer. J. Am. Soc. Nephrol..

[4] Bozack A.K., Hall M.N., Liu X., Ilievski V., Lomax-Luu A.M., Parvez F., Siddique A.B., Shahriar H., Uddin M.N., Islam T. (2019). Folic acid supplementation enhances arsenic methylation: Results from a folic acid and creatine supplementation randomized controlled trial in Bangladesh. Am. J. Clin. Nutr..

[5] Kurzius-Spencer M., da Silva V., Thomson C.A., Hartz V., Hsu C.H., Burgess J.L., O’Rourke M.K., Harris R.B. (2017). Nutrients in one-carbon metabolism and urinary arsenic methylation in the National Health and Nutrition Examination Survey (NHANES) 2003–2004. Sci. Total Environ..

[6] Dubey M., Shea T.B. (2007). Potentiation of arsenic neurotoxicity by folate deprivation: Protective role of S-adenosyl methionine. Nutr. Neurosci..

[7] Centers for Disease Control and Prevention, National Center for Health Statistics (2012). National Health and Nutrition Examination Survey (NHANES) 2011–2012.

[8] Centers for Disease Control and Prevention, National Center for Health Statistics (2014). National Health and Nutrition Examination Survey (NHANES) 2013–2014.

[9] Centers for Disease Control and Prevention, National Center for Health Statistics (2016). National Health and Nutrition Examination Survey (NHANES) 2015–2016.

[10] Centers for Disease Control and Prevention, National Center for Health Statistics (2018). National Health and Nutrition Examination Survey (NHANES) 2017–2018.

[11] Centers for Disease Control and Prevention (CDC), National Center for Health Statistics (NCHS) (2011). National Health and Nutrition Examination Survey Laboratory Procedures.

[12] Centers for Disease Control and Prevention. https://wwwn.cdc.gov/Nchs/Nhanes/2017-2018/BIOPRO_J.htm.

[13] Levey A.S., Stevens L.A., Schmid C.H., Zhang Y.L., Castro A.F., Feldman H.I., Kusek J.W., Eggers P., Van Lente F., Greene T. (2009). A new equation to estimate glomerular filtration rate. Ann. Intern. Med..

[14] Levey A.S., de Jong P.E., Coresh J., El Nahas M., Astor B.C., Matsushita K., Gansevoort R.T., Kasiske B.L., Eckardt K.U. (2011). The definition, classification, and prognosis of chronic kidney disease: A KDIGO Controversies Conference report. Kidney Int..

[15] Tooze J.A., Midthune D., Dodd K.W., Freedman L.S., Krebs-Smith S.M., Subar A.F., Guenther P.M., Carroll R.J., Kipnis V. (2006). A new statistical method for estimating the usual intake of episodically consumed foods with application to their distribution. J. Am. Diet. Assoc..

[16] Yeung L.F., Cogswell M.E., Carriquiry A.L., Bailey L.B., Pfeiffer C.M., Berry R.J. (2011). Contributions of enriched cereal-grain products, ready-to-eat cereals, and supplements to folic acid and vitamin B-12 usual intake and folate and vitamin B-12 status in US children: National Health and Nutrition Examination Survey (NHANES), 2003–2006. Am. J. Clin. Nutr..

[17] Bailey L.B., Stover P.J., McNulty H., Fenech M.F., Gregory J.F., Mills J.L., Pfeiffer C.M., Fazili Z., Zhang M., Ueland P.M. (2015). Biomarkers of Nutrition for Development-Folate Review. J. Nutr..

[18] Pfeiffer C.M., Sternberg M.R., Fazili Z., Lacher D.A., Zhang M., Johnson C.L., Hamner H.C., Bailey R.L., Rader J.I., Yamini S. (2015). Folate status and concentrations of serum folate forms in the US population: National Health and Nutrition Examination Survey 2011–2012. Br. J. Nutr..

[19] Fazili Z., Sternberg M.R., Potischman N., Wang C.Y., Storandt R.J., Yeung L., Yamini S., Gahche J.J., Juan W., Qi Y.P. (2020). Demographic, Physiologic, and Lifestyle Characteristics Observed with Serum Total Folate Differ Among Folate Forms: Cross-Sectional Data from Fasting Samples in the NHANES 2011–2016. J. Nutr..

[20] Pfeiffer C.M., Sternberg M.R., Fazili Z., Yetley E.A., Lacher D.A., Bailey R.L., Johnson C.L. (2015). Unmetabolized folic acid is detected in nearly all serum samples from US children, adolescents, and adults. J. Nutr..

[21] Visentin M., Diop-Bove N., Zhao R., Goldman I.D. (2014). The intestinal absorption of folates. Annu. Rev. Physiol..

[22] Zhao R., Matherly L.H., Goldman I.D. (2009). Membrane transporters and folate homeostasis: Intestinal absorption and transport into systemic compartments and tissues. Expert Rev. Mol. Med..

[23] Kamen B.A., Smith A.K. (2004). A review of folate receptor alpha cycling and 5-methyltetrahydrofolate accumulation with an emphasis on cell models in vitro. Adv. Drug Deliv. Rev..

[24] Crider K.S., Qi Y.P., Devine O., Tinker S.C., Berry R.J. (2018). Modeling the impact of folic acid fortification and supplementation on red blood cell folate concentrations and predicted neural tube defect risk in the United States: Have we reached optimal prevention?. Am. J. Clin. Nutr..

[25] Chen M.Y., Rose C.E., Qi Y.P., Williams J.L., Yeung L.F., Berry R.J., Hao L., Cannon M.J., Crider K.S. (2019). Defining the plasma folate concentration associated with the red blood cell folate concentration threshold for optimal neural tube defects prevention: A population-based, randomized trial of folic acid supplementation. Am. J. Clin. Nutr..

[26] Pfeiffer C.M., Sternberg M.R., Zhang M., Fazili Z., Storandt R.J., Crider K.S., Yamini S., Gahche J.J., Juan W., Wang C.Y. (2019). Folate status in the US population 20 y after the introduction of folic acid fortification. Am. J. Clin. Nutr..

[27] Saydah S.H., Pavkov M.E., Zhang C., Lacher D.A., Eberhardt M.S., Burrows N.R., Narva A.S., Eggers P.W., Williams D.E. (2013). Albuminuria prevalence in first morning void compared with previous random urine from adults in the National Health and Nutrition Examination Survey, 2009–2010. Clin. Chem..

[28] Coresh J., Selvin E., Stevens L.A., Manzi J., Kusek J.W., Eggers P., Van Lente F., Levey A.S. (2007). Prevalence of chronic kidney disease in the United States. JAMA.

[29] Delgado C., Baweja M., Crews D.C., Eneanya N.D., Gadegbeku C.A., Inker L.A., Mendu M.L., Miller W.G., Moxey-Mims M.M., Roberts G.V. (2021). A Unifying Approach for GFR Estimation: Recommendations of the NKF-ASN Task Force on Reassessing the Inclusion of Race in Diagnosing Kidney Disease. J. Am. Soc. Nephrol..

[30] Plantinga L.C., Boulware L.E., Coresh J., Stevens L.A., Miller E.R., Saran R., Messer K.L., Levey A.S., Powe N.R. (2008). Patient awareness of chronic kidney disease: Trends and predictors. Arch. Intern. Med..

[31] Ojodu J., Hulihan M.M., Pope S.N., Grant A.M. (2014). Prevention. Incidence of sickle cell trait--United States, 2010. MMWR Morb. Mortal. Wkly. Rep..

[32] Naik R.P., Derebail V.K., Grams M.E., Franceschini N., Auer P.L., Peloso G.M., Young B.A., Lettre G., Peralta C.A., Katz R. (2014). Association of sickle cell trait with chronic kidney disease and albuminuria in African Americans. JAMA.

[33] Key N.S., Derebail V.K. (2010). Sickle-cell trait: Novel clinical significance. Hematol. Am. Soc. Hematol. Educ. Program..

