# Supplementary material for: Reduced Kidney Function Is Associated with Increasing Red Blood Cell Folate Concentration and Changes in Folate Form Distributions (NHANES 2011–2018)
Source: Nutrients. 2022 Mar 2;14(5):1054. doi: 10.3390/nu14051054 (PMC8912286; doi:10.3390/nu14051054)

Supplementary Materials

Table S1: Concentrations of red blood cell (RBC) folate and serum total folate, RBC/serum ratio, and folic acid usual intake in persons ≥16 y, stratified by albuminuria stages, NHANES 2011–2018 [1].

	Albuminuria stages			
**Analyte**	**A1 (<30 mg/g)**	**A2 (30–299 mg/g)**	**A3 (≥ 300 mg/g)**	**p-value**
Sample Size, n	16021	1768	388	
RBC folate, nmol/L	1103 (1083, 1124)	1204 (1165, 1244)	1285 (1189, 1389)	<0.0001
Serum total folate, nmol/L [2]	37.2 (36.3, 38.1)	40.1 (38.2, 42.1)	36.1 (33.4, 39.1)	0.006
RBC/serum ratio	29.7 (29.2, 30.1)	30.0 (29.1, 31.0)	35.6 (32.3, 39.3)	<0.0001
Usual intake, µg (IQR) [3]	226 (109, 390)	211 (93, 383)	228 (107, 407)	0.18

[1] Values represent the adjusted geometric mean (95% CI), adjusting for body mass index, poverty-income ratio, and education levels; p-values calculated from Wald test within demographic subgroup across albuminuria stages risk groups.

[2] Serum total folate is the sum of folate forms (5-methylTHF, non-methyl folate, folic acid) excluding MeFox.

[3] Values represent the median (interquartile range [IQR]), adjusted for age, poverty-income ratio, race-Hispanic origin, and education.

Table S2: Concentrations of red blood cell (RBC) folate and serum total folate, RBC/serum ratio, and folic acid usual intake in persons ≥16 y, stratified by estimated glomerular filtration rate (eGFR) stages, NHANES 2011–2018.

	eGFR stage					
**Analyte**	**G1 (≥90)**	**G2 (60-90)**	**G3a (45-59)**	**G3b (30-44)**	**G4/G5 (<30)**	**p-value**
**Standard eGFR**						
Sample Size, n	11816	5093	826	352	40	
RBC folate [1]*, nmol/L*	1053 (1035, 1070)	1189 (1162, 1217)	1328 (1270, 1389)	1640 (1519, 1770)	1271 (839, 1924)	< 0.0001
Serum total folate [1,2]*, nmol/L*	35.4 (34.7, 36.2)	40.0 (38.4, 41.6)	44.2 (41.6, 47.0)	53.7 (48.3, 59.6)	26.4 (19.2, 36.4)	< 0.0001
RBC/serum ratio [1]	29.7 (29.3, 30.1)	29.8 (28.9, 30.6)	30.0 (28.7, 31.5)	30.5 (28.5, 32.7)	48.4 (37.4, 62.7)	0.013
Folic acid usual intake, µg (IQR) [3]	216 (108, 366)	241 (109, 428)	228 (103, 407)	254 (104, 464)	175 (82, 317)	0.011
**eGFR, race not in equation** [4]						
Sample Size, n	11074	5603	999	397	54	
RBC folate[5]*, nmol/L*	1061 (1043, 1078)	1167 (1142, 1192)	1297 (1247, 1350)	1585 (1472, 1707)	1406 (990, 1997)	< 0.0001
Serum total folate [2,5]*, nmol/L*	35.5 (34.8, 36.3)	39.5 (38.1, 41.0)	43.3 (40.9, 45.7)	52.4 (47.3, 58.0)	30.5 (22.5, 41.3)	< 0.0001
RBC/serum ratio [5]	29.8 (29.4, 30.3)	29.5 (28.7, 30.3)	30.0 (28.7, 31.3)	30.3 (28.4, 32.3)	46.3 (37.7, 56.9)	0.0072
Folic acid usual intake, µg (IQR) [3]	215 (107, 364)	245 (111, 434)	228 (101, 411)	271 (122, 459)	184 (85, 332)	0.095

[1] Values represent the adjusted geometric mean (95% CI), adjusting for body mass index, poverty-income ratio, and education levels; p-values calculated from Wald test within demographic subgroup across eGFR stages.

[2] Serum total folate is the sum of folate forms (5-methylTHF, non-methyl folate, folic acid) excluding MeFox.

[3] Values represent the median (interquartile range [IQR]), adjusted for age, poverty-income ratio, race-Hispanic origin, and education.

[4] eGFR calculated removing the 1.159 multiplier for non-Hispanic Black individuals.

[5] Values represent the adjusted geometric mean (95% CI) represent the adjusted geometric mean adjusted for body mass index, poverty-income ratio, education levels, and race-Hispanic origin; p-values calculated from Wald test within demographic subgroup across eGFR stages

Table S3: Concentrations of red blood cell (RBC) folate and serum total folate and RBC/serum ratio in demographic subgroups ≥16 y, stratified by chronic kidney disease (CKD) risk group, with additional adjustments, NHANES 2011–2018 [1].

	**CKD Risk Group**				
**Analyte**	**Low Risk**	**Moderate Risk**	**High Risk**	**Highest Risk**	**p-value**
**RBC folate (nmol/L)**					
**Overall**	1092 (1072, 1112)	1196 (1164, 1230)	1457 (1383, 1534)	1455 (1300, 1627)	<0.0001
16-59 y	1054 (1034, 1075)	1068 (1025, 1112)	1274 (1171, 1387)	1287 (967, 1738)	0.0033
60-74 y	1223 (1182, 1265)	1286 (1226, 1350)	1371 (1251, 1504)	1465 (1263, 1700)	0.0033
≥75 y	1382 (1326, 1440)	1467 (1381, 1559)	1715 (1592, 1847)	1540 (1315, 1802)	0.0012
**Serum folate (nmol/L)[2]**					
**Overall**	36.8 (35.9, 37.8)	39.9 (38.6, 41.3)	45.7 (41.7, 50.2)	42.7 (37.6, 48.6)	<0.0001
16-59 y	34.9 (34.1, 35.8)	34.8 (32.8, 37.0)	33.8 (31.1, 36.8)	29.3 (23.3, 36.8)	0.17
60-74 y	43.4 (41.1, 45.7)	42.4 (39.9, 44.9)	40.1 (33.0, 48.7)	39.3 (33.5, 46.2)	0.43
≥75 y	55.2 (52.1, 58.4)	53.2 (48.7, 58.1)	65.6 (58.1, 74.2)	54.2 (44.9, 65.4)	0.034
**RBC/serum ratio**					
Overall	29.6 (29.2, 30.1)	30.0 (29.2, 30.7)	31.9 (29.6, 34.3)	34.1 (31.1, 37.5)	0.0010
16-59 y	30.2 (29.8, 30.6)	30.7 (29.5, 31.9)	37.7 (33.3, 42.7)	44.2 (36.8, 53.1)	<0.0001
60-74 y	28.2 (27.2, 29.2)	30.4 (29.1, 31.7)	34.2 (29.8, 39.3)	37.2 (32.1, 43.2)	<0.0001
≥75 y	25.0 (24.0, 26.0)	27.6 (25.8, 29.5)	26.1 (24.3, 28.1)	28.6 (26.0, 31.4)	0.016

[1] Values represent the adjusted geometric mean (95% CI), adjusting for body mass index, poverty-income ratio, education levels, sex and Hispanic origin; CKD risk determined by eGFR and albuminuria stages as outlined by the Kidney Disease Improving Global Outcomes workgroup; p-values calculated from Wald test within demographic subgroup across CKD risk groups.

[2] Serum total folate is the sum of folate forms (5-methylTHF, non-methyl folate, folic acid) excluding MeFox.

[3] Non-methyl folate is the sum of 3 minor forms: THF, 5-formylTHF, and 5,10-methenylTHF; non-methyl folate is below level of detection (<LOD) if all 3 minor forms were <LOD.

Figure S1: Participant flowchart of NHANES 2011–2018.

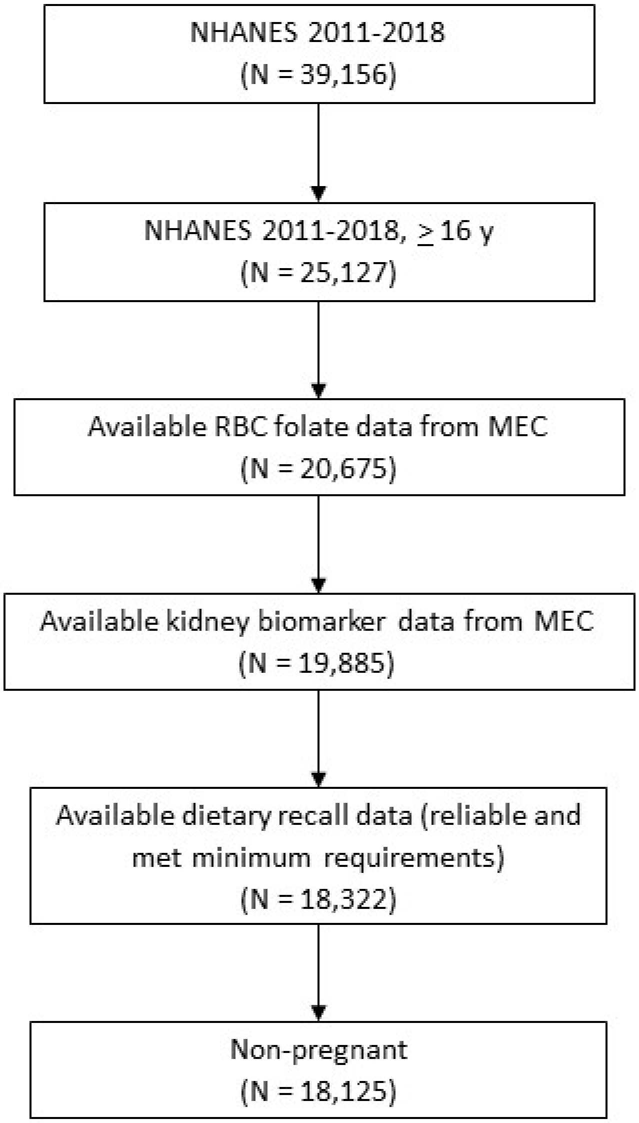

Supplement: Supplementary file 1 [file nutrients-14-01054-s001.zip › nutrients-1593214-supplementary.pdf]
